# Pleomorphic adenoma presenting with conductive hearing loss in the ear canal: a case report and review of the literature

**DOI:** 10.1186/1752-1947-8-178

**Published:** 2014-06-06

**Authors:** Ayako Maruyama, Takao Tokumaru, Ken Kitamura

**Affiliations:** 1 Department of Otolaryngology, Tokyo Medical and Dental University, Bunkyo-ku, Yushima 1-5-45, Tokyo 113-8519, Japan

**Keywords:** Benign tumor, Pleomorphic adenoma, Ear canal

## Abstract

**Introduction:**

Pleomorphic adenoma accounts for 65 percent of all salivary gland tumors. It has been identified in several anatomical regions, but pleomorphic adenoma arising in the ear canal, first described in 1951, is extremely rare.

**Case presentation:**

A 40-year-old Japanese man’s left ear canal was obstructed by a pleomorphic adenoma that caused mild conductive hearing loss. The tumor was resected and he remains disease-free two years after surgery.

**Conclusions:**

Pleomorphic adenoma usually arises from a major and minor salivary gland, but pleomorphic adenoma of the ear canal is derived from the ceruminous gland. We discuss the present case and 37 other case reports in our effort to clarify the clinical features and the course of pleomorphic adenoma in the ear canal.

## Introduction

Pleomorphic adenoma (PA) is a benign tumor that usually originates in a major salivary gland. It also arises from minor salivary glands and other structures in the head and neck region such as the lip and cheek [1]. PA arising in the ear canal is very rare. Since the first report of PA in the ear canal by Mark and Rothberg in 1951 [2], there have been only a few similar case reports. PA in the ear canal is derived primarily from the ceruminous gland, the modified apocrine sweat gland of the ear canal [3]. We report a case of PA arising in the ear canal and provide a brief review of the relevant literature.

## Case presentation

**Figure 1:**
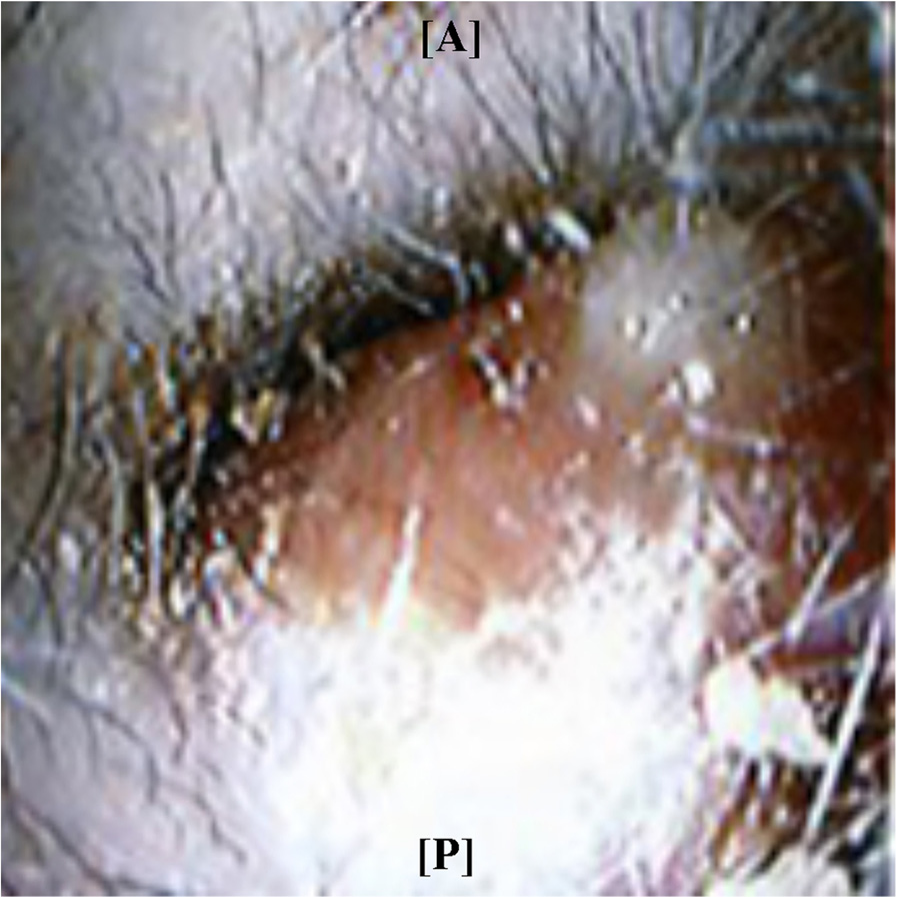
Clinical features of the 40-year-old man at the first visit: the pleomorphic adenoma tumor arises from the posterior wall of his left ear canal.

**Figure 2:**
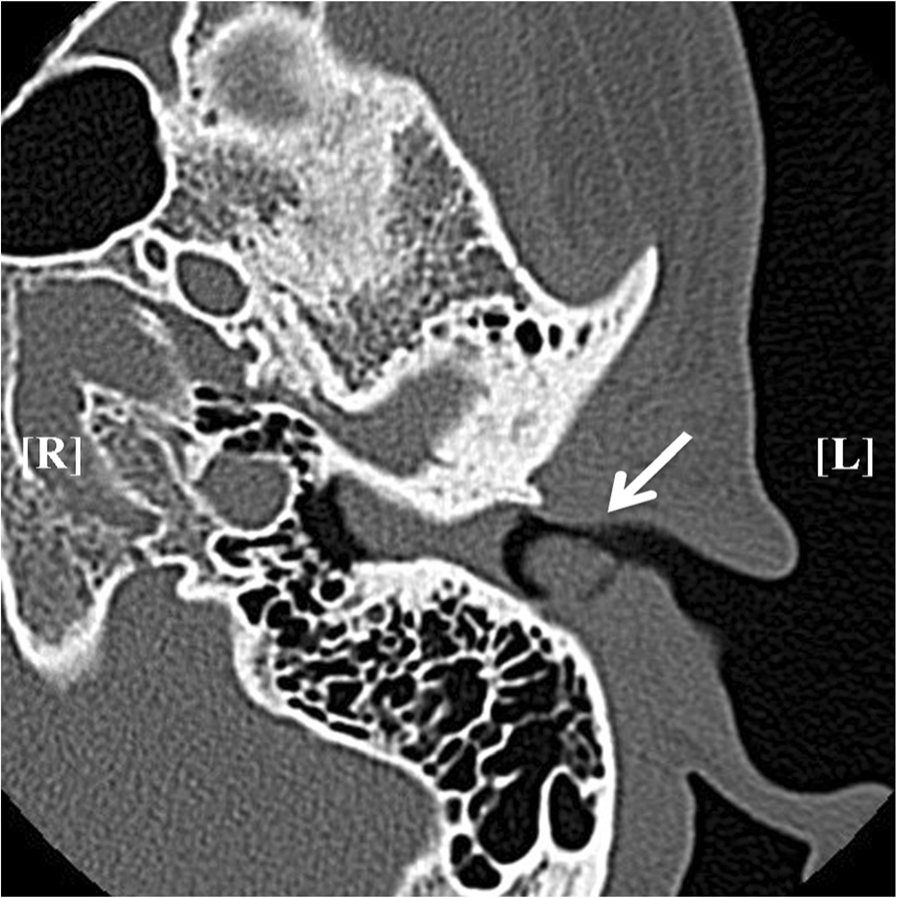
**Computed tomography imaging: computed tomography showing the left ear canal mass (white arrow).** The mass between the tumor and the tympanic membrane was thought to be earwax.

A 40-year-old Japanese man presented at an Ear, Nose and Throat clinic with left-side hearing loss and tinnitus without otalgia or vertigo that had been present in the prior 10 days. A tumor was identified at the opening of his left ear canal, and he was referred to our hospital. The tumor was smoothly covered by the skin, and it was not mobile. It obstructed his ear canal so that the tympanic membrane was hidden from view (Figure 1). The pure tone audiogram showed mild conductive hearing loss with an air-bone gap of 20 to 45dB. Computed tomography showed a homogeneous mass from the posterior wall of his left ear canal without infiltration into the other structures. In the ear canal, the area between the tumor and the tympanic membrane was isodense (Figure 2).

**Figure 3:**
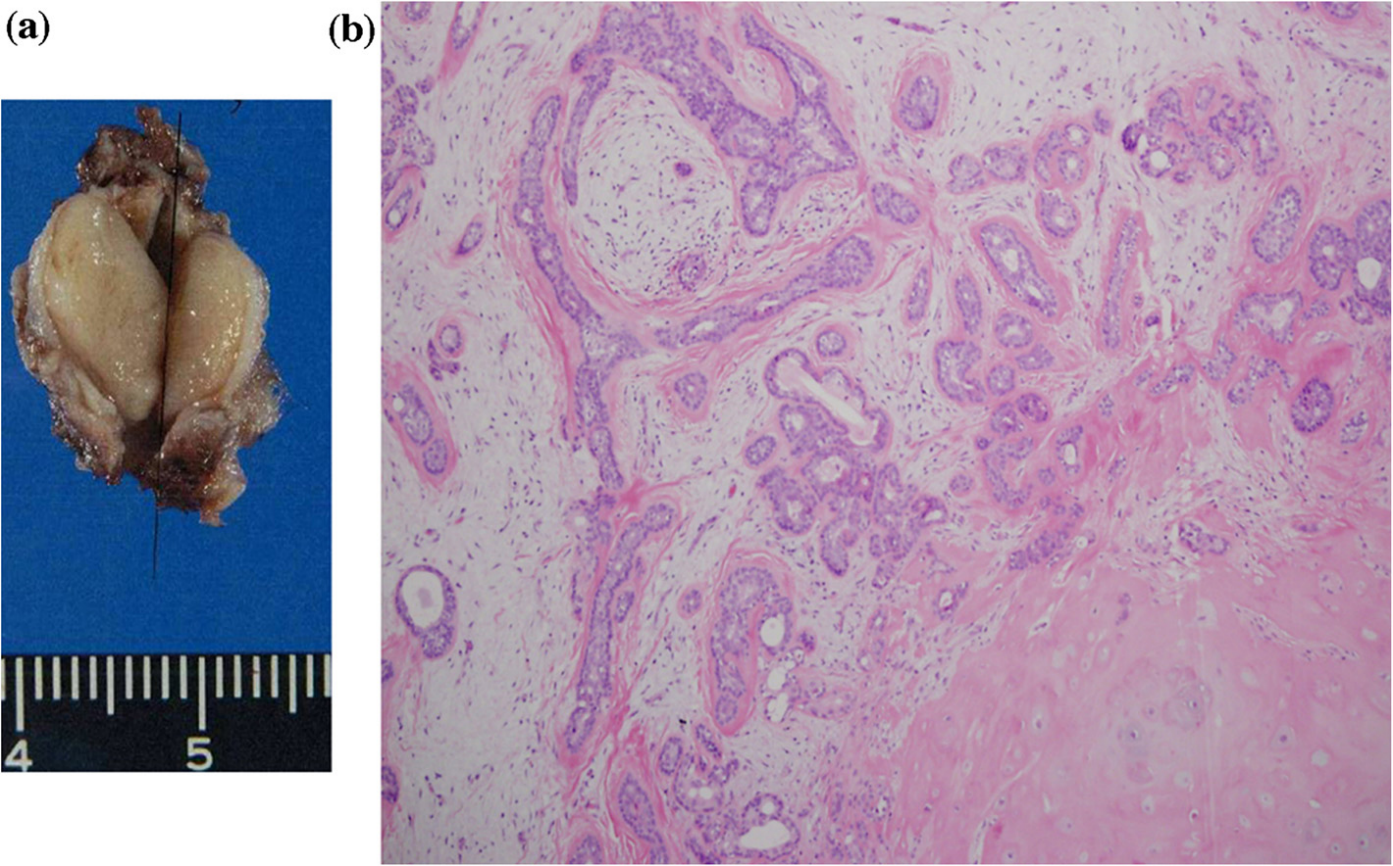
**Resected tumor. (a)** The tumor size was 18×12×12mm, and its cut surface was whitish. **(b)** Histological findings of the tumor showing the glandular epithelial component and mixoid stroma.

Considering these noninvasive findings, this tumor was suspected to be benign. Surgical treatment under general anesthesia was performed two months after the start of symptoms. The tumor was resected *en bloc* via a postaural and endaural approach with a margin including cartilage and skin. A significant amount of hyperkeratotic substances were present behind the tumor, apparently retained debris. The tympanic membrane was preserved without appreciable change. A full-thickness retroauricular skin flap and fascia temporalis were used to cover the cutaneous defect of the ear canal.The tumor size was 18×12×12mm. It was well circumscribed and its cut surface was whitish (Figure 3a). Microscopically, the tumor showed a mixture of epithelial cells with formed ducts and myxomatous stroma with spindle cells. It had a cartilage component. The pathological diagnosis was PA (Figure 3b). The tumor was sharply marginated, but its capsule was not formed sufficiently, so that it invaded the surrounding fat tissue in some places. The margin of the excision was clear. Approximately two years after the tumor removal, there was no stenosis of the ear canal or recurrence of the tumor.

## Discussion

PA, a mixed tumor, accounts for 65 percent of all tumors of the salivary glands [1]. They mostly arise from a major salivary gland, but they can arise at other parts of the head and neck, including the minor salivary glands as well as the cheeks and eyelids. However, PA of the ear canal is very rare. Kuo *et al*. reported 37 cases of PA arising from sites other than the major salivary glands, and only one of the 37 cases was observed in the ear canal [1].

**Figure 4:**
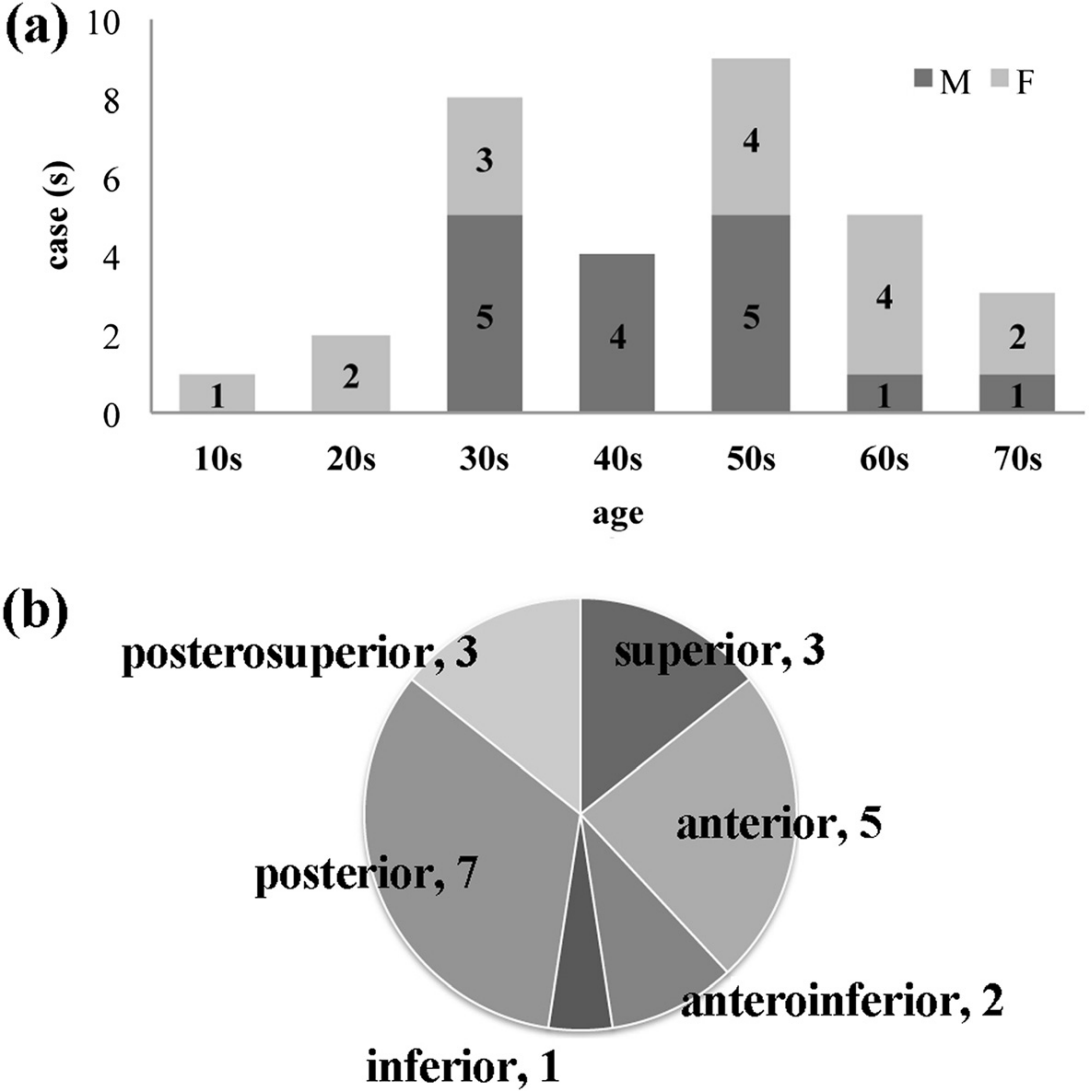
**The age range and tumor sites of similar cases. (a)** The age range of 37 cases. **(b)** Tumor sites in the canal.

Since the first report of a PA arising in the ear canal by Mark and Rothberg in 1951, there have been 37 similar cases reported in the English literature [1-10]. There were 16 males and 16 females (the gender of the other five cases was not mentioned) and their ages ranged from 15 to 79 years (average 46.0 years) (Figure 4a). The tumor sizes varied from 0.5 to 3.5cm. The sites in the canal are presented in Figure 4b. The symptoms of PA arising in the ear canal varied: hearing loss, otalgia, tinnitus, aural fullness, otorrhea, and sometimes no symptoms (Table 1). These symptoms were caused by the obstruction of the ear canal by the tumor, as in cases of benign tumors of the ear canal [11]. The duration of symptoms before clinical presentation ranged from 10 days to 13 years. As with other benign tumors, the duration of symptoms was often a period of years. The cases were sometimes associated with chronic otitis media and cholesteatoma [5,6].

**Table 1:** Symptoms of 37 cases

**Symptoms**	**Case(s)**
Hearing loss	12
Mass	12
Otalgia	8
Aural fullness	5
Otorrhea	5
Tinnitus	4
Itching	2
Headache	2
Pressure	1
Infection	1
Bloody otorrhea	1
Head noise	1
Dizziness	1
Facial palsy*	1
Asymptomatic	2

*A case-associated cholesteatoma presented as facial palsy.

(Some cases had more than one symptom) [1-10]

Historically, PA of the ear canal was called ceruminoma, indicating glandular tumors of the ear canal regardless of whether they were benign or malignant. Welti classified these tumors into four groups: ceruminous adenoma, adenoid-cystic carcinoma, ceruminous adenocarcinoma, and the most rare, pleomorphic adenoma [3]. Based on his classification, syringocystadenoma papilliferum has been added as a benign neoplasm arising from the ceruminous gland [12]. The ceruminous gland is the cerumen-secreting modified apocrine sweat gland located in the cartilaginous portion of the ear canal. In these tumors, it is important to distinguish malignancy. Adenoid cystic carcinoma is the most commonly reported primary ceruminous gland malignancy [13]. Invasiveness appears to be the only sign of malignancy, but the symptoms do not always reflect the character of tumors so Hicks recommended early excisional biopsy [3,14]. At the same time, there is an opinion that these tumors should be taken as potentially malignant because the biopsy is not sufficiently reliable if it is limited [13].

PA of the ear canal is characterized by the presence of a subepithelial proliferation of glandular structures with nests of myoepithelial components in a chondromyxoid stroma, similar to the histology of PA of salivary gland origin [12]. Myoepithelial cells of the ceruminous glands are thought to be the origin of primary PA of the ear canal; they differentiate and can form a component of the tumor [4].

*En bloc* surgical excision with a sufficient margin is important in the treatment of PA of the EAC to prevent recurrence. Recurrence occurs in approximately 5 percent of PAs arising in the parotid gland after primary surgery, caused by capsule rupture, a positive margin, and tumor spillage [15]. Additionally, 3 to 4 percent of PAs arising from a salivary gland may transform into carcinoma ex-pleomorphic adenomas.

In contrast, the malignant transformation of a PA of the ear canal has been reported only by Botha and Kahn, as ‘aggressive chondroid syringoma’ [7]. Chondroid syringoma is the term used in dermatological practice to describe PA from skin appendages [7,8]. In that case, the tumor recurred with cellular atypism and mitotic activity and accompanied several satellite nodules three years after the first resection [7]. Another case of recurrence was reported by Pahor *et al*.; it occurred because of incomplete resection but showed no malignant transformation [9].

Postoperative radiotherapy for PA is controversial. It has been reported as not effective and can induce malignant change, although it has improved the local control for patients with inadequate margins and/or multinodular recurrence [10,15]. Because the effect of radiotherapy may uncertain, it is best to try to avoid the necessity for radiotherapy. A sufficient excision without breaking the capsule is important for obtaining a good prognosis and a long follow-up after surgery is necessary to check for any recurrence.

## Conclusions

We describe a case of PA arising in the ear canal. PA of the ear canal is very rare and it presents with various symptoms. Surgical excision with sufficient margins and a long follow-up are important in the treatment.

## Consent

Written informed consent was obtained from the patient for publication of this case report and any accompanying images. A copy of the written consent is available for review by the Editor-in-Chief of the journal.

## Abbreviations

PA: pleomorphic adenoma.

## Competing interests

The authors declare that they have no competing interests.

## Authors’ contributions

AM designed the clinical review, performed most of the work, did most of the data analysis, and wrote most of the paper. TT helped with the data analysis. KK performed the surgery, helped with the data analysis and wrote parts of the paper. All authors read and approved the final manuscript.
